# Influence of Hole Transport Layers on Buried Interface in Wide-Bandgap Perovskite Phase Segregation

**DOI:** 10.3390/nano14110963

**Published:** 2024-06-01

**Authors:** Fangfang Cao, Liming Du, Yongjie Jiang, Yangyang Gou, Xirui Liu, Haodong Wu, Junchuan Zhang, Zhiheng Qiu, Can Li, Jichun Ye, Zhen Li, Chuanxiao Xiao

**Affiliations:** 1 School of Materials Science and Chemical Engineering, Ningbo University, Ningbo 315211, China; caofangfang@nimte.ac.cn (F.C.); liuxirui@nimte.ac.cn (X.L.); wuhaodong@nimte.ac.cn (H.W.); 2 Ningbo Institute of Materials Technology and Engineering, Chinese Academy of Sciences, Ningbo 315201, China; jiangyongjie@nimte.ac.cn (Y.J.); gouyangyang@nimte.ac.cn (Y.G.); zhangjunchuan@nimte.ac.cn (J.Z.); qiuzhiheng@nimte.ac.cn (Z.Q.); jichun.ye@nimte.ac.cn (J.Y.); 3 State Key Laboratory of Solidification Processing, Center for Nano Energy Materials, School of Materials Science and Engineering, Northwestern Polytechnical University and Shaanxi Joint Laboratory of Graphene (NPU), Xi’an 710072, China; dlm2021@mail.nwpu.edu.cn (L.D.); lican@nwpu.edu.cn (C.L.); 4 Nano Science and Technology Institute, University of Science and Technology of China, Hefei 230041, China; 5 Ningbo New Materials Testing and Evaluation Center Co., Ltd., Ningbo 315201, China

**Keywords:** light-induced phase segregation, perovskite solar cells, hole transport layer, buried interface, microscopy

## Abstract

Light-induced phase segregation, particularly when incorporating bromine to widen the bandgap, presents significant challenges to the stability and commercialization of perovskite solar cells. This study explores the influence of hole transport layers, specifically poly[bis(4-phenyl)(2,4,6-trimethylphenyl)amine (PTAA) and [4-(3,6-dimethyl-9H-carbazol-9-yl)butyl]phosphonic acid (Me-4PACz), on the dynamics of phase segregation. Through detailed characterization of the buried interface, we demonstrate that Me-4PACz enhances perovskite photostability, surpassing the performance of PTAA. Nanoscale analyses using in situ Kelvin probe force microscopy and quantitative nanomechanical mapping techniques elucidate defect distribution at the buried interface during phase segregation, highlighting the critical role of substrate wettability in perovskite growth and interface integrity. The integration of these characterization techniques provides a thorough understanding of the impact of the buried bottom interface on perovskite growth and phase segregation.

## 1. Introduction

Wide-bandgap perovskite solar cells (PSCs) offer great advantages, including a high absorption coefficient [1], tunable bandgap [2,3,4,5], and long carrier diffusion length [6,7,8], making them suitable as top cells in tandem solar cell configurations. In the perovskite ABX_3_ structure, incorporating other ions at the A-site (e.g., MA^+^, Rb^+^) and X-site can effectively regulate the perovskite lattice structure, achieving high bandgap perovskite materials and broaden the spectral response of tandem solar cells [9,10,11,12]. At present, most of the top sub-cells in high-efficiency tandem solar cells are prepared by adjusting the bromine-to-iodine ratio [13,14]. However, a significant challenge during their operation is the occurrence of light-induced phase segregation, which severely compromises the stability of PSCs. To widen the bandgap, a high percentage of bromine (up to 40%), mixed with iodine, is commonly employed. These two halide ions can be unstable, leading to their segregation into distinct phases. Such phase segregation not only forms defects but also significantly impacts the open-circuit voltage (V_OC_) and causes substantial degradation [15]. This issue limits the further advancement of PSC-based tandem cells. Understanding the mechanism of phase segregation is crucial for enhancing the stability of wide-bandgap perovskite solar cells and facilitating the commercialization of tandem cells.

Currently, strategies to inhibit phase segregation primarily focus on increasing grain sizes, passivating grain boundaries and interface defects [16,17,18,19,20,21], and optimizing charge transport layers [22]. Achieving a uniform and dense perovskite/hole transport layer (HTL) interface is essential for obtaining high efficiency and fill factor in perovskite solar cells. The interface between the HTL and perovskite film can harbor numerous defects due to nonideal contact, leading to the formation of iodine vacancies and gap states [23,24,25]. The properties of HTL materials significantly impact the composition uniformity and charge extraction efficiency at the buried interface of the perovskite film. This results in insufficient charge extraction and significant nonradiative recombination [26,27,28,29]. To address this, self-assembled monolayer (SAM) materials with superior wettability have been introduced to replace the commonly used HTL material poly[bis(4-phenyl)(2,4,6-trimethylphenyl)amine (PTAA), resulting in more uniform perovskite films [30,31,32,33]. The Π-Π conjugation effect of aromatic amines in SAM materials enhances the interaction between the HTL layer and the perovskite layer, leading to the formation of a higher-quality perovskite film and interface, which exhibits excellent hole extraction performance [29,34]. Among these, the molecular design of HTL to effectively improve hole extraction has emerged as a promising approach to inhibit phase segregation and improve stability [35,36,37]. Zhang [38] et al. incorporated conjugated linkers and intramolecular donor–acceptor (D-A) groups to stabilize electron-rich arylamines through effective electron/charge delocalization and energy level modulation. These conjugated D-A structures enhance the photo and electrical stability of organic transport materials. Al-Ashouri [11] et al. proposed and confirmed that phase separation can be suppressed when perovskite is grown on a substrate, such as [4-(3,6-dimethyl-9H-carbazol-9-yl)butyl]phosphonic acid (Me-4PACz), that meets the requirements of rapid charge extraction and effective passivation. The SAMs based on carbazole can create passivated interfaces while minimizing transport losses due to their ultrathin nature. Despite advancements in phase segregation inhibition [39,40], the focus has primarily been on characterizing the upper surface, with limited attention given to the buried interface. However, the critical issue may lie within the buried bottom interface, which is often challenging to expose. Therefore, characterizing the buried bottom interface is essential for a deeper understanding of the degradation and improvement mechanisms.

In this work, we explored the influence of HTLs on the properties and phase segregation of perovskite films. By carefully peeling off the perovskite layer, we elucidated the topographical, electrical, and mechanical properties of perovskite films at the buried interface and investigated the process of halide ion migration under illumination. The use of Me-4PACz as the HTL significantly enhanced the photostability and carrier lifetime of the perovskite materials. Solar cells with Me-4PACz as the HTL demonstrated superior efficiency and a reduced hysteresis effect compared to those using PTAA as the HTL. In situ Kelvin probe force microscopy (KPFM) scanning of the peeled-off side provided a nanometer-scale resolution topography and defect distribution before and after illumination. Additionally, PeakForce Quantitative Nanomechanics (QNM) mapping identified a softer defective region associated with phase segregation. The integration of these characterization techniques offers a comprehensive understanding of how the buried bottom interface influences perovskite growth and phase segregation.

## 2. Experimental Section

### 2.1. Materials

All materials used in this study were commercially available. Glass/ITO (8 Ω sq^−1^) was purchased from Liaoning You Xuan New Energy Technology Co., Ltd. (Dalian, China) Lead (II) iodide (PbI_2_, 99.99%), lead (II) bromide(PbBr_2_, 99.99%), and [4-(3,6-Dimethyl-9H-carbazol-9-yl)butyl] (Me-4PACz) were acquired from TCI Shanghai Chemical Industry Materials Corp. (Shanghai, China). Dimethyl formamide (DMF, 99.8%, anhydrous), dimethyl sulfoxide (DMSO, 99.9%, anhydrous), ethyl acetate (EA, 99.8%, anhydrous), 2-propanol (IPA, anhydrous, 99.5%), poly [bis (4-phenyl) (2,4,6-triMethylphenyl)amine] (PTAA), and lead thiocyanate (Pb(SCN)_2_, 99.5%) were obtained from Sigma-Aldrich (St. Louis, MO, USA) without further purification. Formamidinium iodide (FAI, 99.99%) was purchased from Greatcell Solar Materials Pty Ltd. (Queenstown, New South Wales, Australia). And 2-ThEABr was bought from Xi’an Polymer Light Technology Corp. (Xi’an, China).

### 2.2. Perovskite Film Fabrication

The perovskite film possesses a bandgap of 1.78 eV and consists of approximately 40% bromine and 60% iodine. We incorporated a 10% excess of lead iodine into the precursor, following a preparation method similar to that outlined in refs [41,42]. The precursor solution (1.2 M) was formulated from four precursors dissolved in a mixed solvent of DMF and DMSO with a volume ratio of 4:1. Then, 4.85 mg (1.5 mol% relative to Pb) of Pb(SCN)_2_ was introduced into the solution. The molar ratios for CsI/FAI/PbI_2_/PbBr_2_ were 0.8:0.2:0.4:0.6. The structure of the perovskite film includes glass/ITO/HTL/perovskite, where the HTLs were PTAA and Me-4PACz. Glass/ITO was cleaned in an ultrasonic bath in the sequences of detergent solution, deionized water, acetone, and isopropanol for 15 min in each step. To enhance the wettability of PTAA and Me-4PACz, we employed DMF (100 μL) and IPA (100 μL) to continue to clean the ITO substrate for 15 min, and then the samples were treated with UV–ozone for 15 min before spin coating. The perovskite precursor solution was thoroughly mixed at 50 °C for 2 h before usage. The PTAA (2 mg/mL) was dissolved in chlorobenzene, spin-coated onto the ITO at a speed of 5000 rpm, and then annealed at 100 °C for 10 min. Me-4PACz (1.00 mg/mL) solution dissolved in isopropanol was spin-coated on the ITO at 5000 rpm for 15 min. Finally, the perovskite precursor solution was spin-coated at 5000 rpm for 30 s with 0.2 mL of EA dripped slowly at the last 10 s. And we annealed the as-deposited films at 100 °C for 10 min. On the finished cells, C60 (30 nm), BCP (5 nm), and Ag (120 nm) were sequentially deposited by thermal evaporation.

### 2.3. Exposure of Buried Bottom Interface

We directly peeled off the perovskite film to expose the buried bottom interface. First, a drop of UV glue (ThreeBond, 3055B, Tokyo, Japan) was applied onto the perovskite surface, which was rapidly covered with another piece of ITO glass. This was crucial to ensure the absence of air bubbles and the even spread of the UV glue. Additionally, the cover ITO glass should not be perfectly aligned with the target peel-off sample, leaving space to facilitate the separation of the two glasses. Subsequently, the UV glue was exposed to a 365 nm UV lamp for 180 to 300 s until it was fully cured. Once the UV glue had solidified, the two glasses were separated by applying manual force in opposite directions, resulting in the clean removal of the perovskite film. This procedure is illustrated in Figure S1. Note that the roughness of the peel-off glass may not affect the results. For example, the more commonly used fluorine tin oxide (FTO) has a much rougher topography than ITO, and perovskites grown on ITO and FTO substrates may exhibit different nucleation and crystallinity processes. However, the crystal growth of the perovskite film did not occur on the FTO glass. And during the peeling-off process, UV glue was applied, which eliminated the roughness differences. The ITO or FTO glass was used as a conductive substrate and may not affect the sequence studies.

### 2.4. Characterizations

The PL and atomic force microscopy (AFM) measurements were conducted inside a glovebox environment with water and oxygen levels maintained below 0.01 ppm (as indicated by the glovebox meter). PL measurements were conducted using a homemade system equipped with a fiber optic spectrometer (Ocean Optics, QE Pro, Shanghai, China). A laser with a wavelength of 532 nm and a power of 200 mW was employed for excitation. The I–V measurements were conducted on unencapsulated inverted wide-bandgap perovskite cells with an effective area of 3 mm × 1.5 mm, irradiated by AM 1.5G light with an intensity of 100 mW·cm^−2^ from a solar simulator (Sol3A 94063A, Newport, Beijing, China). The measurements were calibrated using a KG-5 filtered silicon standard solar cell (SRC-2020, NREL calibrated). I–V curves were recorded using a source meter (Keithley 2400, Shanghai, China) at a scan rate of 0.1 V·s^−1^ in both reverse and forward directions, with a voltage scan range from −0.1 V to 1.4 V. KPFM and QNM measurements were carried out using a Bruker Dimension Icon AFM system. The KPFM system utilized a home-built setup designed to generate the Kelvin probe signal by continuously nullifying the electrostatic force between the probe and the sample. The probe utilized in KPFM was PPP-EFM from Nanosensor, while QNM measurements employed a DDESP-V2 probe from Bruker. To eliminate the possible artifact in KPFM measurement caused by rough topography, we used a probe with a high spring constant (Bruker RTESPA-525, k = 200 N/m) to scratch away materials and make a dent and we observed no significant change in both topography and surface potential mappings before and after light soaking.

## 3. Results and Discussion

**Figure 1:**
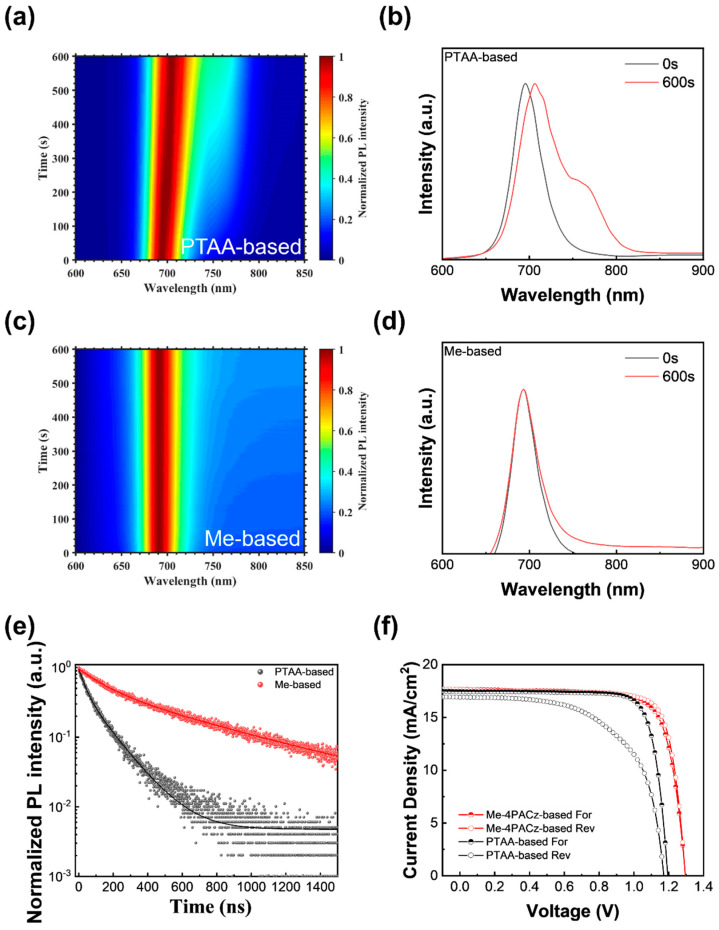
(**a**) In situ photoluminescence (PL) of perovskite materials grown on PTAA; (**b**) the corresponding PL spectra at 0 and 600 s under laser illumination; (**c**) in situ PL of perovskite materials grown on Me-4PACz; (**d**) the corresponding PL spectra at 0 and 600 s under laser illumination; (**e**) time-resolved PL of the two perovskite samples; (**f**) current–voltage characteristics of cells fabricated from the two perovskite samples.

We investigated phase stability and performance differences between wide-bandgap perovskite films grown on Me-4PACz and PTAA substrates with the perovskite materials composed of FA_0.8_Cs_0.2_PbI_1.8_Br_1.2_. Initially, the photoluminescence (PL) peak for films on PTAA substrates was centered at 695 nm, corresponding to a bandgap of 1.78 eV, which aligns with the literature findings [41]. Upon subjecting the samples to 600 s of continuous 532 nm laser illumination, a slight redshift to 705 nm was observed, indicating the emergence of iodine-rich perovskite with lower bandgaps, while the bromine-rich perovskite remained relatively stable (Figure 1a). In contrast, Me-4PACz-based perovskite samples exhibited minimal changes in PL spectra under identical conditions (Figure 1c). Comparative analysis of PL spectra profiles before and after laser excitation was shown in Figure 1b,d. Initially, the full width at half maximum (FWHM) of the PTAA–perovskite sample was 40.14 nm, with no additional peaks observed other than at 695 nm. However, following 600 s of laser illumination, the PTAA–perovskite sample displayed pronounced shoulders at longer wavelengths, whereas the Me-4PACz–perovskite sample exhibited only a slightly wider spectrum width with an FWHM from 36.09 nm to 37.78 nm. These findings underscore the superior phase stability of perovskite films grown on Me-4PACz substrates. Similarly, the PL peak of the PTAA–perovskite sample exhibited a redshift after white LED light soaking for two hours, while the Me-4PACz–perovskite sample showed minimal change, as illustrated in Figure S2.

The Me-4PACz-grown perovskite outperformed the PTAA-grown counterpart in terms of carrier lifetime and cell performance. Time-resolved photoluminescence (TRPL) results indicated a longer carrier lifetime for the Me-4PACz-based sample (detailed fitted parameters are shown in Table S1), and nonradiative recombination was suppressed between Me-4PACz and the perovskite film (Figure 1e). And current–voltage (I–V) measurements of representative cells based on these HTLs revealed performance discrepancies. The PTAA-based cells exhibited an efficiency of 11.94%, with a V_OC_ of 1.17 V, a short-circuit current (J_SC_) of 16.92 mA/cm^2^, and a fill factor (FF) of 60.21%. In contrast, Me-4PACz-based cells demonstrated efficiency of 18.03%, with a V_OC_ of 1.30 V, a J_SC_ of 17.66 mA/cm^2^, and an FF of 78.81%, as well as a smaller hysteresis effect. The detailed forward and reverse scan results are shown in Table S2. The primary improvement in efficiency was attributed to the better HTL/perovskite interface, which reduced halide ion migration during phase segregation and resulted in fewer defects with nonradiative recombination.

We hypothesize that substrate-induced phase segregation contributes to the observed differences. To investigate this, we focused on variations in wetting properties induced by different substrates. PTAA, known for its hydrophobic nature, displayed inferior wettability, as confirmed by water contact angle measurements, as shown in Figure S3. This inferior wetting property could potentially impact perovskite film growth. For direct observation, we peeled off the film to expose the buried bottom interface of the perovskite and assessed its photostability by scanning in the same location after light soaking. We then examined the electrical properties and potential distribution by surface potential mapping via KPFM and assessed nanomechanical properties through QNM mapping.

**Figure 2:**
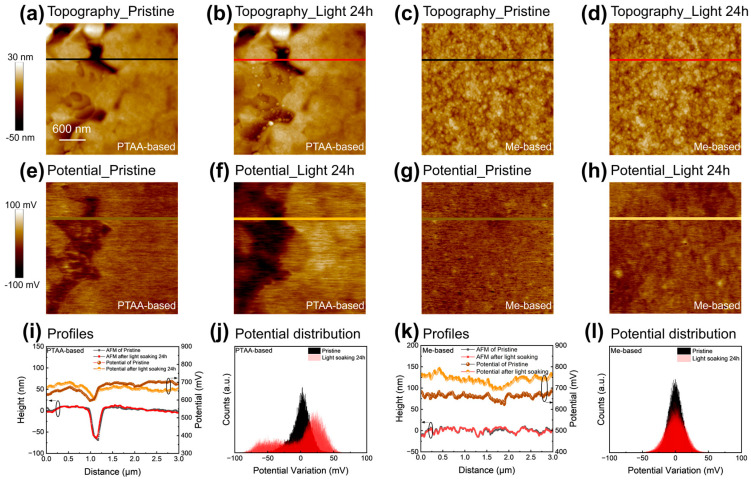
KPFM results on the same location before and after light soaking for 24 h. (**a**,**b**,**e**,**f**) Topography and surface potential of the buried interface of perovskite films grown on PTAA before and after light soaking. (**c**,**d**,**g**,**h**) Topography and surface potential of the buried interface of the perovskite film grown on Me-4PACz before and after light soaking. (**i**) The topography and surface potential profiles of PTAA-based and (**k**) Me-4PACz-based perovskite film. The surface potential distribution of (**j**) PTAA-based and (**l**) Me-4PACz-based perovskite film.

Analysis of the surface potential mapping revealed distinct differences between perovskite films grown on the PTAA and Me-4PACz substrates. Scanning random areas confirmed that perovskite materials grown on PTAA exhibited dents in topography imaging, with the regions around the dents displaying lower potential (Figure 2a,e). It is important to note that the rough topography around the dent regions did not affect the surface potential measurements, as indicated in Figure S4. Subsequent KPFM results (Figure 2b,f) in the same location after 24 h of light soaking revealed small particles (a few tens of nanometers) appearing in the dent regions. These particles altered the roughness of the buried interface, potentially leading to higher interfacial recombination. Importantly, we observed an increase in contrast between high-potential and low-potential regions after light soaking (Figure 2i). Particularly, the regions with new particles exhibited even lower potential values. This lower potential suggests the presence of higher work function materials, such as iodine-rich perovskite, indicating the migration of iodine ions toward the void regions under the influence of light. This migration results in a higher fraction of iodine in the mixed bromine-iodine perovskite materials, leading to nonuniform surface potential distribution due to the redistribution of ions. Quantitative analysis revealed that the roughness R_q_ of surface potential increased from 15.6 mV to 24.7 mV after light soaking (Table S3).

In contrast, the perovskite materials grown on Me-4PACz exhibited a much more uniform potential distribution (Figure 2g). Light soaking had no discernible effect on the topography, as no new particles were observed (Figure 2c,d). Moreover, the surface potential showed negligible changes in both its absolute value and spatial distribution (Figure 2k,l), with the roughness of the surface potential R_q_ changing only from 12.4 mV to 16.3 mV. The fact that we peeled off the perovskite film and directly probed the bottom of the perovskite suggests that the more stable film grown on Me-4PACz HTL indicates the use of self-assembled single-molecule materials can better regulate the nucleation and growth process of the perovskite, resulting in a more uniform perovskite layer. This less defective perovskite film effectively suppressed photo-induced phase segregation.

**Figure 3:**
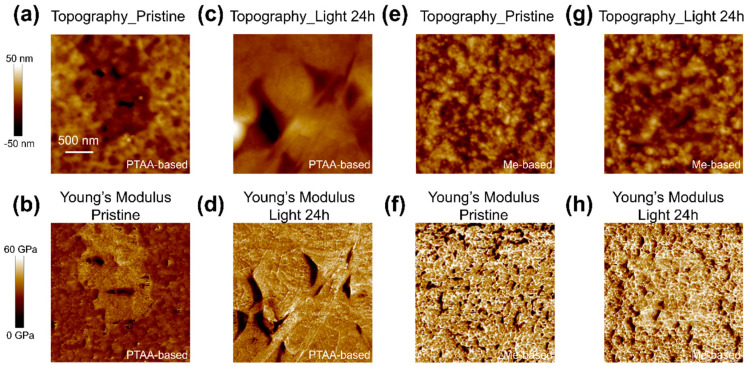
Quantitative nanomechanical measurements on representative areas. (**a**–**d**) Topography (**top**) and stiffness (**bottom**) of the buried interface of perovskite films grown on PTAA before and after light soaking for 24 h. (**e**–**h**) Topography (**top**) and stiffness (**bottom**) of the buried interface of the perovskite film grown on Me-4PACz before and after light soaking for 24 h.

We further investigated the nanomechanical properties of the buried surface of the perovskite and found evidence of changes in stiffness under phase segregation. The results are shown in Figure 3. QNM is an AFM-based technique that maps the material’s stiffness and calculates Young’s modulus. Before illumination, the perovskite films grown on PTAA exhibited Young’s modulus in the range of 30 GPa (Figure S5a), consistent with previously reported results indicating that perovskite is a soft material [43]. The low stiffness suggests that the material may undergo changes under slight mechanical force or be more prone to ion motion and phase changes under external stress such as light [44]. The dents on the PTAA-based perovskite film showed smaller Young’s modulus values, indicating weaker points that are more susceptible to ion migration. Furthermore, the difference between Young’s modulus in the dented area and the surrounding area continued to increase after illumination.

In general, a smaller ionic radius results in a shorter bond length and a stronger binding force. Hence, the mechanical strength reflects lead–halide bonds, with the lead–bromine (Pb-Br) bond being stiffer than the lead–iodine (Pb-I) bond. The lower Young’s modulus in QNM mapping suggests a higher prevalence of Pb-I bonds than Pb-Br bonds and vice versa [44,45,46,47]. In this case, light-induced ion migration causes more iodine ions to move toward the dent regions, forming more Pb-I bonds, which in turn replace Pb-Br bonds. Therefore, the Young’s modulus around the dent regions becomes even smaller. The inference that more iodide ions migrate to the dents after illumination is consistent with the KPFM results in Figure 2. On the other hand, the buried interface of the Me-4PACz-based perovskite film exhibits a uniform distribution of Young’s modulus. The Young’s modulus values are concentrated at around 40 GPa (Figure S5b), which is higher than that of the PTAA-based perovskite film. After illumination, there was no significant change in Young’s modulus, indicating no obvious light-induced ion migration at the buried interface.

Our findings underscore the critical role of substrate choice in perovskite film growth and phase stability. Enhanced wettability of HTL materials at buried interfaces can promote homogeneous film formation, inhibiting light-induced phase segregation. The dents shown in Figure 2 and Figure 3 likely represent defective regions at the buried interface, where high nonradiative recombination occurs. Ion migration induced by light tends to accumulate at the dent regions. Iodine ions have high mobility, with binding energy as low as 0.1 eV [48], making them prone to movement under the influence of light. The segregation of iodine and bromine leads to the formation of more defective sites, ultimately resulting in lower V_OC_ and FF in device performance. In this work, the PL spectra indicate the segregation of iodine- and bromine-rich materials, while microscopic mappings reveal weak points from the perspective of surface potential and nanomechanical properties. In contrast, perovskite materials grown on Me-4PACz have fewer dents at the interface and tend to be more homogenized. The presence of aromatic amines in Me-4PACz makes halide ions easier to fix, thereby enhancing the binding force at the interface between HTL and perovskite [38]. These results provide new perspectives for studying phase segregation. The quality of perovskite film growth is essential for phase stability, and characterizing such a buried interface is challenging. Our approach of peeling off the film and mapping the same location provides unambiguous support for a deeper understanding of the phase segregation mechanism in wide-bandgap perovskite materials.

## 4. Conclusions

In this study, we characterized the influence of ion migration during photo-induced phase separation at the buried interface of inverted wide-bandgap perovskite films. By employing KPFM scanning at the same location, we could directly observe the influence of different HTLs on the buried interface. A significant accumulation of holes was observed at the buried interface of the PTAA-based perovskite film, with a pronounced accumulation of iodine ions through ion migration channels activated by light. In contrast, due to its uniform buried interface, the Me-4PACz-based perovskite film prevented halide ion migration, thereby inhibiting phase separation and achieving enhanced performance and light stability. Our QNM results indicated that the Young’s modulus at the hole was smaller than in other regions, with an increased accumulation of Pb-I bonds at the hole. Upon illumination, an increased migration of iodine ions to the holes resulted in a rise in the Young’s modulus of the surrounding area. Notably, the buried interface of the Me-4PACz-based perovskite film exhibited an almost constant Young’s modulus distribution before and after illumination, indicating minimal ion migration on the buried interface of the Me-4PACz perovskite film. Our new approach, which combines film peeling with nanometer-scale electrical and mechanical property characterization, provides important insights into the phase segregation mechanism. By focusing on the buried interface, this methodology could be employed to further investigate the quality of other interfaces and various degradation modes.

## Data Availability

Data are contained within the article and Supplementary Materials.

## Supplementary Materials

The following supporting information can be downloaded at: https://www.mdpi.com/article/10.3390/nano14110963/s1, Figure S1. The process for peeling off the perovskite film and exposing the buried bottom interface. Figure S2. The PL tests of (a) PTAA-based and (b) Me-4PACz-based perovskite films before and after light soaking for 2 hours. Table S1. The fitting parameters of TRPL decay for the PTAA-based and Me-4PACz-based perovskite films. Table S2. Parameters of PTAA-based and Me-4PACz-based PSCs’ performance. Figure S3. Water contact angle images of (a) PTAA-based and (b) Me-4PACz-based perovskite films. Figure S4. KPFM scans on other areas of the buried interface from PTAA-based and Me-4PACz-based wide bandgap perovskite films. Table S3. The roughness information of PTAA-based and Me-4PACz-based buried interface of perovskite films. Figure S5. Young’s modulus line profiles of the (a) PTAA-based and (b) Me-4PACz-based perovskite films before and after illumination.
