# Supplementary material for: Influence of Hole Transport Layers on Buried Interface in Wide-Bandgap Perovskite Phase Segregation
Source: Nanomaterials (Basel). 2024 Jun 1;14(11):963. doi: 10.3390/nano14110963 (PMC11173573; doi:10.3390/nano14110963)
Supplement: Supplementary file 1 [file nanomaterials-14-00963-s001.zip › nanomaterials-3001948-supplementary.pdf]

Supporting Materials

Influence of Hole Transport Layers on Buried Interface in Wide-Bandgap Perovskite Phase Segregation

Fangfang Cao [1,2,†], Liming Du [3,†], Yongjie Jiang [2,4], Yangyang Gou [2], Xirui Liu [1,2], Haodong Wu [1,2], Junchuan Zhang [2,4], Zhiheng Qiu [2,4], Can Li [3], Jichun Ye [2], Zhen Li [3,*] and Chuanxiao Xiao [2,5,*]

[1]School of Materials Science and Chemical Engineering, Ningbo University, Ningbo 315211, China
[2]Ningbo Institute of Materials Technology and Engineering, Chinese Academy of Sciences, Ningbo 315201, China
[3]State Key Laboratory of Solidification Processing, Center for Nano Energy Materials, School of Materials Science and Engineering, Northwestern Polytechnical University and Shaanxi Joint Laboratory of Gra-phene (NPU), Xi'an 710072, China
[4]Nano Science and Technology Institute, University of Science and Technology of China, Hefei 230041, China
[5]Ningbo New Materials Testing and Evaluation Center Co., Ltd., Ningbo 315201, China
[6] These authors contributed equally: Fangfang Cao, Liming Du.
*Correspondence to: lizhen@nwpu.edu.cn; cxiao@nimte.ac.cn

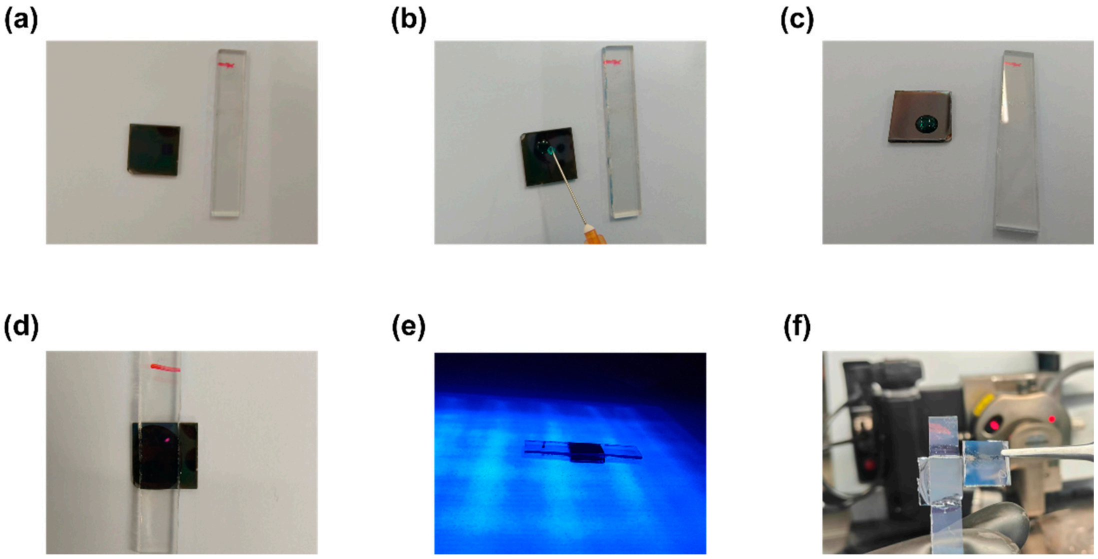

Figure S1. The process for peeling off the perovskite film and exposing the buried bottom interface. (a) Prepare perovskite films and UV glue; (b-c) Apply a drop of UV glue to the perovskite surface; (d) Place an ITO glass over the perovskite film and press evenly; (e) Irradiate the UV glue with a 20W UV lamp for 3-5 minutes; (f) Gently detach the perovskite sample from the ITO glass. Subsequently, the perovskite film is peeled off and adhered to the ITO glass.

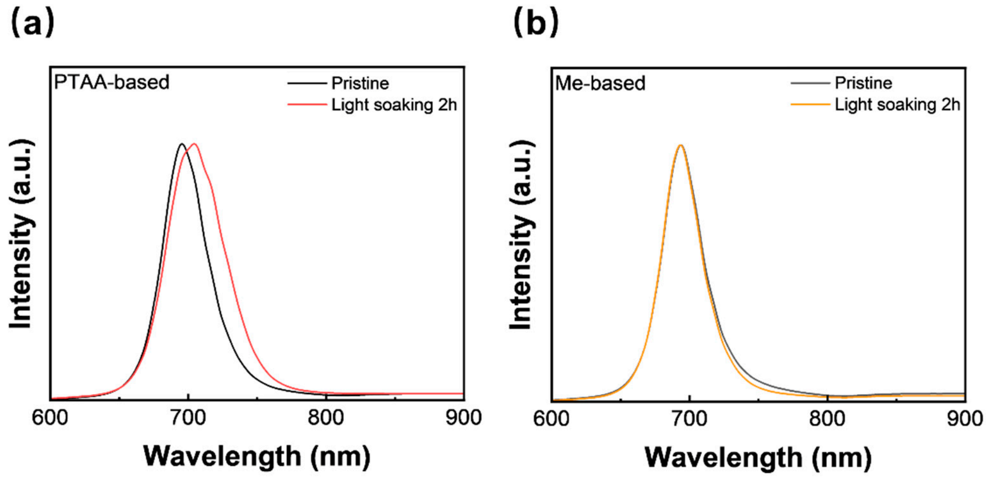

Figure S2. The PL tests of (a) PTAA-based and (b) Me-4PACz-based perovskite films before and after light soaking for 2 hours.

Table S1. The fitting parameters of TRPL decay for the PTAA-based and Me-4PACz-based perovskite films.

Sample	A_1	T_1 (ns)	A_2	T_2 (ns)	T_{ave} (ns)
PTAA-based	0.51	37.8	0.47	137.35	114.46
Me-4PACz-based	0.49	141.96	0.53	612.19	529.18

Table S2. Parameters of PTAA-based and Me-4PACz-based PSCs' performance.

		V_{OC} (V)	J_{SC} (mA/cm^2)	FF (%)	PCE (%)
PTAA-based	Forward	1.19	17.45	79.72	16.61
	Reverse	1.17	16.92	60.21	11.94
Me-4PACz-based	Forward	1.30	17.66	76.56	17.52
	Reverse	1.30	17.66	78.81	18.03

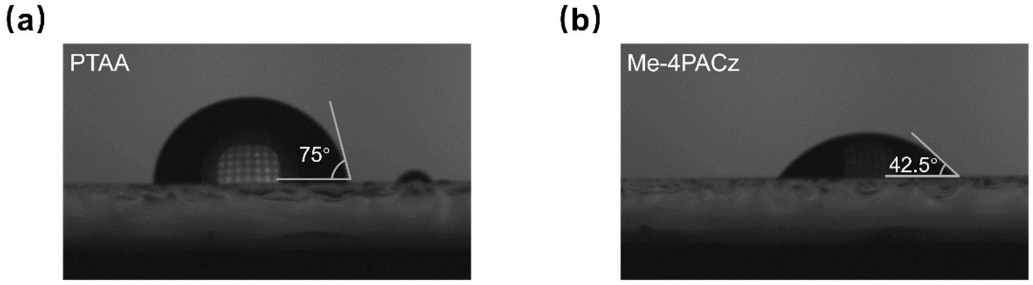

Figure S3. Water contact angle images of (a) PTAA-based and (b) Me-4PACz-based perovskite films.

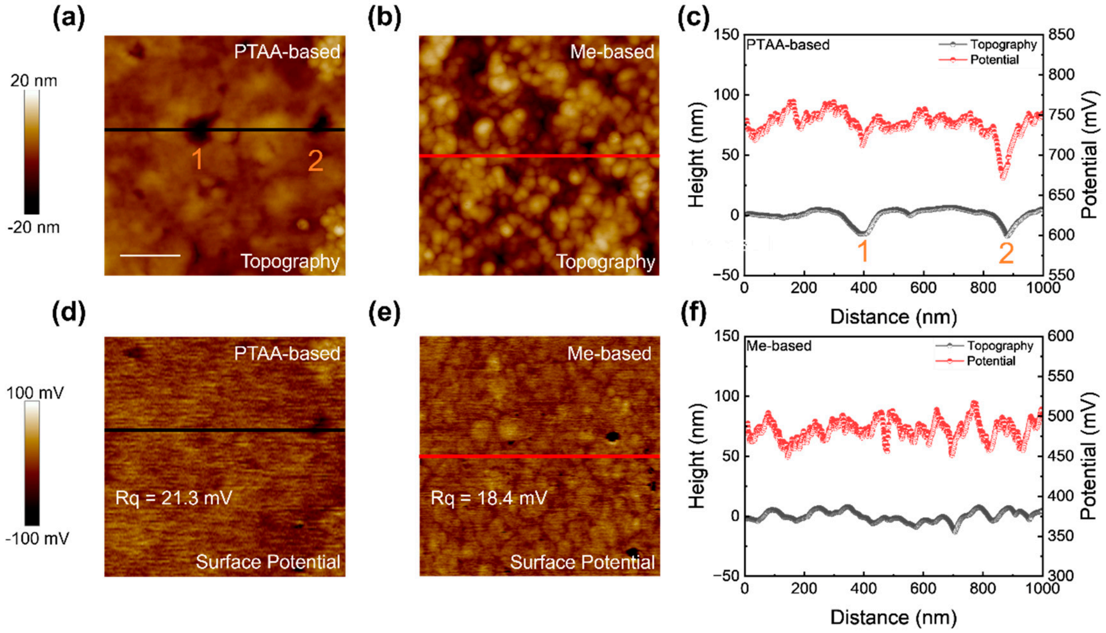

Figure S4. KPFM scans on other areas of the buried interface from PTAA-based and Me-4PACz-based wide bandgap perovskite films. Topography images of the buried interface for (a) PTAA-based and (b) Me-4PACz-based perovskite films reveal dent areas. Corresponding surface potential images are presented in (d) and (e), while (c) and (f) compare the height and potential profiles. The dent regions do not significantly affect the surface potential, as indicated by the "1" and "2" regions. Scale bar: 500 nm.

Table S3. The roughness information of PTAA-based and Me-4PACz-based buried interface of perovskite films.

Sample	AFM R_q (nm)	AFM R_a (nm)	Potential R_q (mV)	Potential R_a (mV)
PTAA-based	9.80	6.09	15.6	11.7
PTAA-based, light 24h	9.93	6.59	24.7	19.4
Me-4PACz - based	5.53	4.51	12.4	9.59
Me-4PACz-based, light 24h	5.75	4.67	16.3	12.9

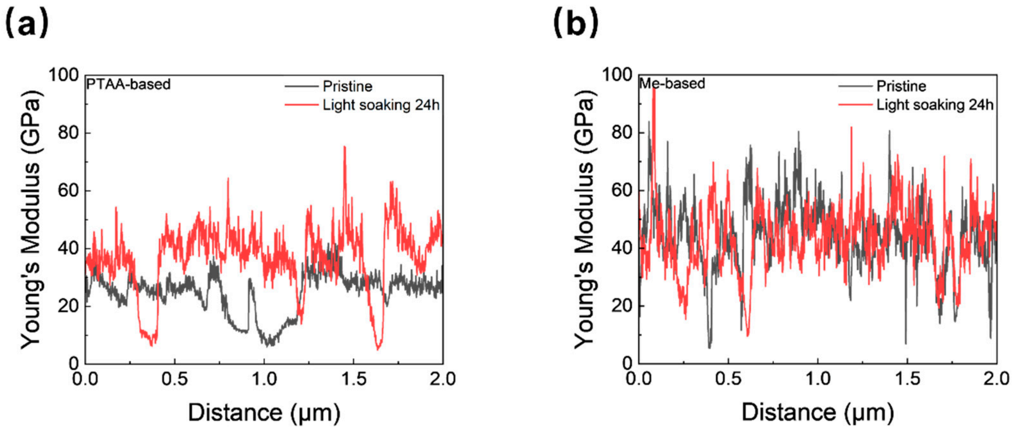

Figure S5. Young's modulus line profiles of the (a) PTAA-based and (b) Me-4PACz-based perovskite films before and after illumination.
